# Analysis of Pesticide Residues by QuEChERS Method and LC-MS/MS for a New Extrapolation of Maximum Residue Levels in Persimmon Minor Crop

**DOI:** 10.3390/molecules27051517

**Published:** 2022-02-23

**Authors:** Pilar Sandín-España, Miguelina Mateo-Miranda, Carmen López-Goti, Elena Seris-Barrallo, José-Luis Alonso-Prados

**Affiliations:** Unit of Plant Protection Products, INIA—CSIC, Ctra. La Coruña, km. 7.5, 28040 Madrid, Spain; mmmateo@inia.es (M.M.-M.); lgoti@inia.es (C.L.-G.); seris.elena@inia.es (E.S.-B.)

**Keywords:** persimmon (*Diospyrons kaki*), pesticides, multi-residue analysis, QuEChERS, LC-MS/MS, MRL extrapolation, minor crop

## Abstract

According to EU guidance SANCO/7525/VI/95 Rev. 10.3, residue data extrapolation from a surrogate major crop to a minor crop can be used for setting maximum residue levels (MRLs) with a reduced number of residue trials and representative selected pesticides. In this work, a QuEChERS method (citrate-buffered version and PSA with MgSO_4_ clean-up) and LC-ESI-MS/MS for the determination of boscalid, pyraclostrobin, fludioxonil, fluopyram and tebuconazole in persimmon was developed and validated according to EU Commission guidelines and afterwards used for the determination of residues in four field trials. Residue levels at harvest for each pesticide ranged between 0.347 and 0.028 mg/kg. After comparing EFSA residue data on apples, as the surrogate major crop, and conducting a consumer risk assessment, a proposal of residue data extrapolation to set MRLs in persimmons was performed. The results showed that pesticide residues in persimmons at harvest were consistently lower than residues in apples when substances were applied according to the same critical GAP. MRLs were set at 0.5 mg/kg for fludioxonil, 0.6 mg/kg for boscalid, 0.3 mg/kg for tebuconazole, 0.4 mg/kg for fluopyran and 0.3 mg/kg for pyraclostrobin. The ratio of the MRLs for apple/persimmon varied between 2.5 for boscalid and 1.25 for fluopyram, suggesting that residue extrapolation can be feasible, promoting the process of pesticide registration for minor crops and the settlement of MRL.

## 1. Introduction

Pesticide residues represent one of the most important factors of chemical contamination in fruits and vegetables. Therefore, maximum residue levels (MRLs) for pesticides in foodstuffs are established in most countries to guarantee consumer safety and to regulate international trade. The MRLs are defined as the upper legal level of a concentration for a pesticide residue in or on food or feed set in accordance with Regulation 396/2005 [1] based on good agricultural practice and the lowest consumer exposure necessary to protect vulnerable consumers. At a global level, the Codex Alimentarius Commission, created in 1963 by the FAO and WHO, sets MRL recommendations worldwide that are not mandatory but aim to improve international trade. In Europe, Commission Regulation (EC) No. 396/2005 [1] was adopted to unify European legislation, to ensure safe food consumption and to avoid differences in MRLs for pesticides between Member States that could involve barriers to trade. In this context, our group, the Unit of Plant Protection Product of INIA-CSIC, is involved in the assessment of the scientific documentation for the evaluation of active substances, plant protection products and setting MRL in Europe as an independent assessing organism in Spain authorized by the Spanish Ministry of Agriculture, Fishery and Food.

Currently, the legislative requirements for the registration of plant protection products are becoming stricter to ensure human and environmental safety [2,3], and the availability of pesticides to be used on crops is becoming more and more limited, compromising the implementation of adequate pest and disease management programs. This situation is critical in the case of “minor crops”, where the main limitation for the registration of pesticides is the establishment of European MRLs, an essential requirement according to Regulation (EC) No 1107/2009 [4]. A crop is classified as ‘minor’ in the European Union when the daily intake contribution is <0.125 g/kg body weight/day or has a cultivation area < 20,000 ha or a production < 400,000 tons per year [5].

The setting of MRLs involves the development of supervised field residue trials that make the cost/benefit unfavorable for these crops. The absence of available pesticides for minor crops is one of the high-priority issues of pesticide residue research [6]. As a consequence, less available pesticides can lead to a misuse of pesticides and the appearance of pest resistances [7].

According to EU guidance SANCO/7525/VI/95 Rev. 10.3 [5], this situation could be solved via the adoption of residue data extrapolation from a surrogate major crop to a minor crop for setting MRLs, using data from field trials performed on the major crop with pesticides used in both crops. This procedure implies the performance of a reduced number of residue trials in the minor crop with representative pesticides to validate the extrapolation. MRLs should be set at levels of residues consistent with the Good Agricultural Practice (GAP) after a consumer risk assessment is performed, taking into account the daily intake of the fruit and the toxicological reference value for the pesticide residue. Therefore, the selection of the surrogate major crop for the extrapolation is crucial to guarantee that levels of residues can be comparable at harvest in both crops or at least that those found in the minor crop do not represent a worse case compared with the levels of residues in the major crop. If the levels of residues are comparable and the consumer risk assessment for the minor crop is acceptable, residue data extrapolation can be set for other pesticides.

Persimmon is classified by guideline SANCO/7525/VI/95 Rev. 10.3 [5] as a minor crop in all of Europe, but no residue data extrapolation is currently set for this crop. The Japanese persimmon (*Diospyros kaki* L. f.), hereafter simply called persimmon, is a fruit native to China, with this country being the biggest producer followed by Korea and Japan, although most of its production is for internal consumption. After these countries, Spain is the fourth producer and presents the greatest and fastest increase of persimmon production among the Mediterranean countries [8]. The selection of the cultivar ‘Rojo Brillante’ with outstanding quality and the removal of astringency without losing fruit firmness represents a substantial improvement in marketing and transport over long distances. This allowed the expansion of persimmon culture in the 1990s. Currently, Spanish production is expected to have been 450,000 tons in 2021 [9], with its distribution mainly concentrated in two areas, the Valencia province and the Andalucía region. Most of this production is exported mainly to the European market.

In this work, the residues of selected pesticides (boscalid, pyraclostrobin, fludioxonil, fluopyram and tebuconazole) in persimmon were investigated in order to establish an extrapolation from a major crop to a minor persimmon crop.

To achieve this goal, crop field trials and residue analysis were conducted to investigate the residue fate of the pesticides in persimmon fruits. In this sense, few methods were found in the literature for the analysis of these pesticides in persimmon, and few residue analyses in persimmon field samples have been performed [10,11]. Furthermore, to the best of our knowledge, no method for the determination of the fungicide fluopyram in persimmon fruit has been previously reported. Therefore, we developed and validated a rapid and accurate method to analyze the pesticides by LC-MS/MS. Considering the sample preparation, different approaches of the QuEChERS method that had not previously been tested were optimized for the extraction of pesticides from the fruit. Afterwards, MRLs were calculated, and a consumer risk assessment according to the European procedure was performed. Based on the results, a residue extrapolation from a major to a minor persimmon crop was proposed.

## 2. Results and Discussion

### 2.1. Optimization of Analytical Method

The extraction step was optimized testing two versions of QuEChERS (quick, easy, cheap, effective, rugged and safe); acetate (Association of Official Agricultural Chemists (AOAC))- and citrate (European Standard (EN))-buffered versions and two different clean-up kits were compared. A total of 3 replicates at a fortification level of 0.1 µg/kg were tested. The recoveries obtained for each compound with the different buffered extraction versions and the clean-up kits of the QuEChERS method are shown in Table 1. As can be observed, good results with recoveries between 89.2 and 103.1% with a relative standard deviation (RSD) in the range of 4.1–10.2% were obtained for all compounds. This is in agreement with the results obtained by other authors [12,13,14], where no significant differences were observed between the different QuEChERS methods, neither in the extraction nor the clean-up kits tested. Therefore, based on the recovery results, no selection can be made. However, it seems that the citrate-buffered QuEChERS version with PSA and MgSO_4_ gave cleaner extracts than the acetate-buffered version, with a higher signal response and less chromatographic interferences for fludioxonil and fluopyram. Therefore, the citrate-buffered and PSA version was selected for subsequent validation and analysis of samples.

To optimize LC separation and peak shape, the effects of variables such as the column, column temperature, and elution conditions were evaluated. Results showed that the Atlantis T3 column provided higher responses, better resolution and a better signal/noise (S/N) ratio for all the compounds studied. Different buffer constituents as their mobile phase and at different pH were tested: (a) 10 mM ammonium formate + 0.1% formic acid; (b) 4 mM ammonium formate + 0.1% formic acid; (c) 0.1% formic acid; (d) 0.2% acetic acid; and € 4 mM ammonium formate (neutral pH). The best signal-to-noise responses for all the compounds studied were obtained with 10 mM ammonium formate + 0.1% formic acid (pH~3) and acetonitrile. These results are in agreement with Min et al. (2011) [11], who optimized the analysis of pyraclostrobin and tebuconazole by LC-MS/MS in different fruits using the same mobile phase. Lee et al. (2012) [10] also observed that this mobile phase gave the optimum results in a multi-residue method to determine 61 pesticides in 4 fruit commodities, including boscalid, tebuconazole and fludioxonil. The best sensitivity and separation were obtained with a flow rate of 0.7 mL/min and a column temperature of 20 °C. The optimized gradient program is described in Section 3. Under the described chromatographic conditions, a good separation can be obtained in a short separation time of 7.5 min.

The ESI-MS/MS conditions selected were in positive mode for all compounds except for fludioxonil, for which the best response was in the negative ionization mode. The product ion of each compound was selected, and the collision energy for each ion transition was optimized. Quantification was performed using the most abundant ion transition, whereas the less abundant ion transition was used for identification. Table 2 summarizes the most relevant MS operating parameters, as well as the MRM transitions selected for quantification and for confirmatory purposes.

**Figure 1:**
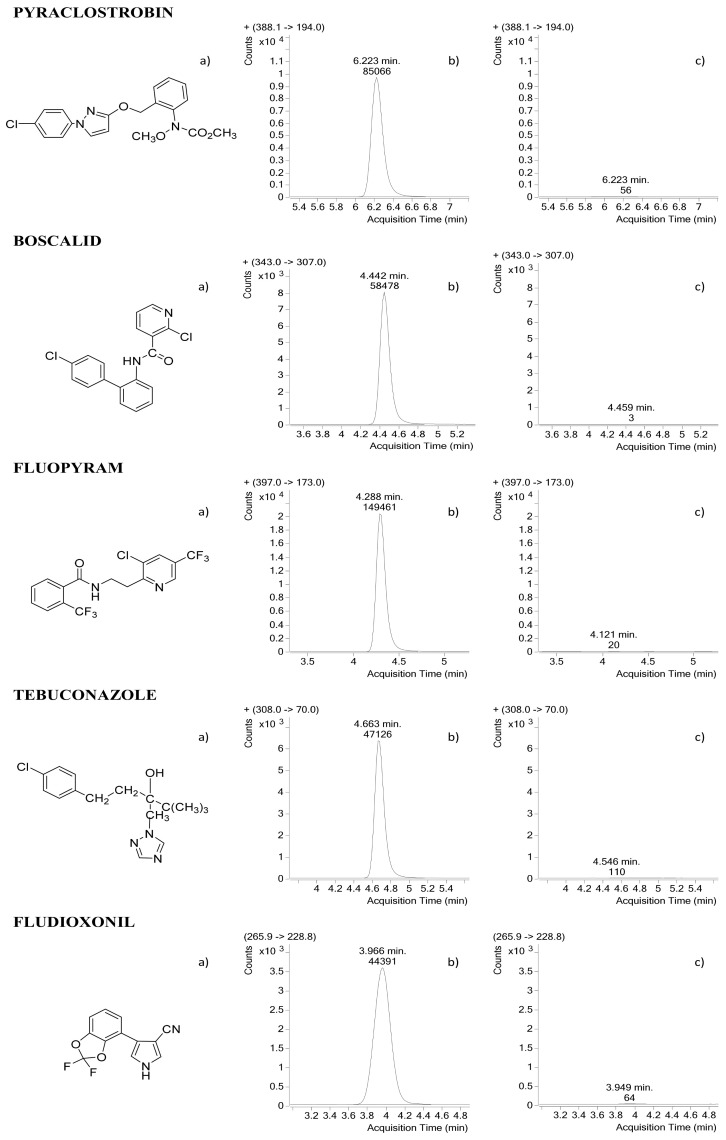
Chemical structures (**a**) and LC-MS/MS chromatograms (quantifier transitions) of the pesticides studied in blank persimmon samples (**b**) and at harvest (**c**).

In the case of fludioxonil, the most abundant ion in the full MS spectrum had a mass of 266, corresponding to an adduct with NH_4_^+^, selecting this ion for fragmentation. This occurs when the mobile phase is ammonium formate and formic acid, and it is in accordance with the work of other authors [10,15,16]. The quantifier ion, 229, was the most abundant product ion, and it can be the result of the loss of the NH_4_^+^ ion and an F atom (loss of 37) (Figure 1). The less abundant ion used for identification was the product ion 158 that could be generated after the loss of the NH_4_^+^ and the pyrrole moiety with the cyano group (loss of 108).

### 2.2. Method Validation

The performance of the developed multi-residue LC-MS/MS method was evaluated according to the SANCO guideline 825/00 [17] of the European Union on the analytical methods for pesticide residues.

To study the well-known matrix effect in an LC analysis [18,19,20], the slopes in solvent solutions were compared to the slopes in matrix-matched solutions. The ratio of the slopes for the solvent and matrix was determined to provide information about the enhancement (Ss/Sm < 1) or suppression (Ss/Sm ˃ 1) of the signal in the matrix. The results are shown in Table 3. Regarding boscalid and tebuconazole, a mild effect was observed, as the Ss/Sm values were close to 1. An enhancement effect was observed on the signal for fludioxonil with a ratio of 0.77, whereas for fluopyram and pyraclostrobin, a suppressive effect was observed with values of 1.29 and 1.24, respectively. Therefore, to compensate for the matrix effect and to avoid any over- or underestimation of residues, a matrix-matched calibration was used to obtain more accurate quantification.

Linearity in matrix-matched solutions was determined covering a working range of 5 points from 1.0 µg/kg to 30 µg/kg (1.0–2.5–10–15–30) for fludioxonil and from 0.7 µg/kg to 15 µg/kg (0.7–1.0–2.5–10–15) for the rest of the compounds, obtaining high correlations for all the target analytes with coefficients of determination (R^2^) > 0.99 (Table 3).

Recoveries and repeatability of the developed analytical method were determined by the analysis of 5 replicate samples (n = 5) at 2 fortification levels corresponding to the LOQ and 10 × LOQ (Table 4). Two untreated unfortified control samples were also analyzed. The results showed satisfactory mean recoveries for all compounds, ranging from 87.65% to 102.01%, with good relative standard deviation values lower than 9.57% in all cases. These results are in accordance with the European Union guidelines SANCO/3029/99 [21], SANCO/825/00 [17] and SANTE/11813/2017 [22] that state that the average recovery should fall in the range of 70–120% with an associated RSD less than or equal to 20%. Method sensitivity was determined by limits of detection and limits of quantification. Limits of detection were calculated as the minimum concentration of an analyte giving a peak with a signal-to-noise ratio of at least 3:1 (Table 3). The validated LOQs were defined as the lowest spike level (expressed in µg/kg) for which a recovery in the 70-120% range could be obtained, with a corresponding RSD ≤ 20%, according to the previously cited guidelines. The LOQ for all the compounds was 1 µg/kg, except for fludioxonil, for which it was 2.5 µg/kg.

The repeatability of the chromatographic method was determined by the relative standard deviation (RSD). The intraday RSD was evaluated by analyzing six replicates of the sample extracts spiked with the active substances at two concentration levels (LOQ and 10 × LOQ) on the same day. The interday RSD was determined by analyzing the compounds in the same sample on three separate days. For both concentrations, the analytes presented an intraday RSD in the range from 2.08% to 6.19% and from 2.19% to 6.57% for the interday RSD (Table 4). These results confirmed that our LC-MS/MS method has satisfactory reliability and repeatability for the quantification of our targeted pesticides in the persimmon matrix.

The developed method in this work improves the determination of these fungicides in persimmon when compared to other methods reported in the literature. Lee et al. (2012) [10] used the AOAC QuEChERS approach to determine boscalid, fludioxonil, pyraclostrobin and tebuconazole in persimmon with acceptable recoveries, except for pyraclostrobin, which was higher than 120%. Furthermore, the LOQ obtained in this work presented LOQ values 10 times lower than those in the cited work. In the case of fluopyram, we obtained a LOQ of 1.0 µg/kg and a LOD of 7 × 10^−3^ µg/kg. To the best of our knowledge, this is the first time that a method has been developed for the determination of this fungicide in persimmon.

### 2.3. Field Results

The previously developed method was applied to the determination of the selected pesticides in four Spanish persimmon cultivars. Table 5 summarizes the content of active substances in the samples analyzed 0 days after the last application (0DALA) and at harvest.

Procedural recoveries were always determined together with the actual analyzed samples to check the extraction efficiency of the method. No pesticide residue was detected in plot A in the four trials used as controls. No residues of pesticides were found in plots where they were not applied, demonstrating that drift from adjacent plots did not occur. In all cases, concentrations detected on the day of application (0DALA) were higher than the amount found at harvest, showing the degradation or dissipation of the active substances in the environment. The RSDs of the residue in analyzed samples in each plot were low in all the analyses, ranging from 0.52% to 3.04%. To our knowledge, there are few studies in the literature that analyze these pesticides in field persimmon samples. Min et al. (2011) [11] developed a method using HPLC-MS/MS for the determination of pesticide residues in different fruits, including persimmon, and analyzed 10 blind-incurred samples. They found pyraclostrobin at a concentration of 0.007 mg/kg lower than the quantities found in our study (0.066–0.089 mg/kg at harvest) and tebuconazole at a concentration of 0.08 mg/kg, which is within the range of our results (0.028–0.137 mg/kg). They did not detect boscalid in the samples of persimmon studied.

### 2.4. Extrapolation Proposal

Considering the residues analyzed for each substance at harvest, the MRLs in persimmons were calculated using the internationally adopted OECD MRL Calculator [23]. MRLs calculated for persimmons were compared with MRLs reported in the reasoned opinions of EFSA about the revision of MRLs in apples for each of these substances (Table 6).

Considering the MRL values derived from these datasets, the ratios between the MRLs for apples and persimmons vary between 2.50 for boscalid and 1.25 for fluopyram. The results show that residues in apples are consistently higher than residues in persimmons when the substances are applied according to the same critical GAPs. Taking into account that the setting of MRL must not pose an unacceptable risk to human health, the assessment of dietary exposure to pesticide residues is a key step in the establishment of these MRLs. Therefore, the acute and chronic risks for consumers should be taken into account. To perform this risk assessment, the estimated dietary exposure is compared with the relevant toxicological reference values, that is, acceptable daily intake (ADI) for the chronic risk assessment and the acute reference dose (ARfD) for the acute risk assessment. Estimated dietary exposure is determined by the residue level in the fruit (mg/kg) and the consumption, and it is expressed by the following equation:

Dietary Exposure = (Residue Concentration × Food Consumption)/Body Weight

The risk is considered acceptable provided that the exposure of the pesticide does not exceed the ADI or the ARfD.

Furthermore, according to data considered in the Pesticide Residue Intake Model (PRIMo) [24], the dietary intake contribution of persimmon is always well below the intake contribution of apples in the 37 diets in either the whole of the EU or in national populations considered in the model. The ratio of the average consumption for apple/persimmon varies between 77 for a Spanish diet and 1248 for a German child’s diet (Table 7).

Considering that the estimated dietary exposure of an active substance is proportional to the residue concentration and the food consumption, no exceedance of ADI or ARfD is expected when data from apples are used for persimmons. Therefore, consumer safety will be guaranteed when setting MRLs in persimmons based on the residue data gathered in apples.

## 3. Materials and Methods

### 3.1. Selection of Crops and Pesticides

Following guideline SANCO/7525/VI/95 Rev. 10.3, different factors that determine the residue behavior of pesticides in crops have been studied in order to find an appropriate major crop susceptible to be used for extrapolation. Apples are classified as a major crop and has multiple similarities with persimmons, such as crop architecture (crop height, foliage densities, and leaf and fruit sizes) as well as crop development, e.g., both crops are perennial fruit crops and deciduous trees with a spring flowering period and harvest in late summer. All these features make apple crops a good candidate to be selected as the surrogate major crop.

Furthermore, according to the EPPO database, there are 17 pests and diseases that are common for apples [25] and persimmons [26]. Therefore, it can be assumed that the same active substances could be applied to control the same pest and disease in both crops.

A total of five active substances were selected based on the different behaviors in the plant, such as systemic and contact activities, as representatives of a broader range of active substances and considering the availability of residue trials in apples. The selected active substances were boscalid, fluopyram and tebuconazole with systemic activities and fludioxonil and pyraclostrobin with contact activities.

### 3.2. Field Trial

Two different locations—in representative growing areas for persimmons—in the East and South of Spain were selected for the study. Two orchards in Seville were selected, one each in the municipalities of Lora del Rio and Villa Nueva del Rio (trials 1 and 2). Another two orchards were also selected in Valencia in the municipality of Alginet (trials 3 and 4). The trials were performed from September to November during the fruiting season. Each trial consisted of a control plot (plot A) and three treated plots (plots B, C and D) without replication. Each plot had a size between 40 and 77 m^2^ and a medium tree height of 3 m. The planting distance was approximately 2.5 × 5.0 m. Each plot of persimmons contained ≥5 trees. Weather data during application were recorded with minimum temperatures between 11 and 17 °C and maximum temperatures of 20–30 °C.

Plots B, C and D were treated with the following products, respectively, by foliar application according to Good Agriculture Practice (GAP): Bellis (Boscalid 25.2% *w*/*w* and pyraclostrobin 12.8% *w*/*w*) water-dispersible granules (WG), Geoxe 50 WD (fludioxonil 50% *w*/*w*) water-dispersible granules (WG) and Luna Experience (fluopyram 17.8% *w*/*w* and tebuconazole 17.8% *w*/*w*) suspension concentrate (SC). The application doses were the following: boscalid, 3 applications of 200 g a.s./ha 1500 L/ha; pyraclostrobin, 3 applications of 100 g a.s./ha 1500 L/ha; fludioxonil, 3 applications of 240 g a.s/ha 1500 L/ha; fluopyram, 4 applications of 150 g a.s/ha 1500 L/ha; tebuconazole, 3 applications of 150 g a.s/ha 1500 L/ha. EFSA reasoned opinions reported the critical GAPs for the major crop apple of boscalid [27], pyraclostrobin [28], fludioxonil [29], tebuconazole [30] and fluopyram [31]. The GAP used in the residue trials performed on the minor crop, persimmon, was identical to the critical GAP considered in the major crop, apple.

The field trials were conducted following different guidelines covering the design, preparation and realization of residue [5,32].

Samples were collected from the untreated and treated plots on the day of the last application (0DALA) and at harvest time. Each specimen was collected randomly from a minimum of 12 different places within each plot. Thus, 12 persimmon fruits were collected randomly in each plot weighing ~3 kg. Samples were placed into individual plastic bags labeled with detailed information. All specimens were stored frozen at a temperature of −18 °C and shipped deep-frozen by a freezer truck to the laboratory, where samples were kept frozen until analysis.

### 3.3. Analytical Determination

#### 3.3.1. Reagents and Chemicals

Analytical standards for the pesticides boscalid (99.9%), pyraclostrobin (99.9%), fludioxonil (99.9%), fluopyram (99.9%) and tebuconazole (99.3%) were supplied by Sigma-Aldrich (Steinheim, Germany) (Figure 1). Two QuEChERS methods were compared for the initial extraction step, including the acetate-buffered version (DisQuE product) supplied by Waters (Milford, MA, USA) and the citrate-buffered EN Standard Method supplied by Biotage, HPC Standards GmbH (Symta, Spain).

For d-SPE clean-up, two products were compared: the DisQuE 2 mL tube–AOAC (900 mg anhydrous MgSO_4_ and 150 mg primary secondary amine (PSA)) and the DisQuE 2 mL tube-EN/C18 (150 mg anhydrous MgSO_4_ and 25 mg (PSA)/25 mg C18), both of which were purchased from Waters (Milford, MA, USA). A total of 3 octadecylsilane (C18) reversed-phase columns of different dimensions, particle sizes and pore sizes were tested: Nova-Pak C18, (4 µm, 150 × 3.9 mm), Atlantis C18 T3 (3.0 µm, 4.6 × 150 mm) and Kinetex C18 (2.6 µm, 100 × 4.6 mm).

Individual stock solutions containing 50 µg/mL of each analyte were prepared in pesticide-grade acetonitrile. The standard solutions were stored at 4 °C in the dark and were used to prepare more diluted standard solutions for calibration and for the recovery study.

Blank persimmon samples used for recovery studies and matrix-matched calibration were purchased from a local organic food store (Madrid, Spain) and were previously checked for the presence of pesticides. As expected, no detectable pesticide residues were found.

#### 3.3.2. Sample Preparation

For the development of the method, 1 kg blank persimmons were chopped and homogenized for 2 min in a Foss HM 2097 food processor. Then, 10 g ± 0.3 of the homogenized samples were weighed into a 50 mL PTFE centrifuge tube, and the standard solution of analytes was added as previously described. Samples and standards were carefully mixed and allowed to stand for 15 min before extraction to allow the pesticides to penetrate into the matrix.

**Figure 2:**
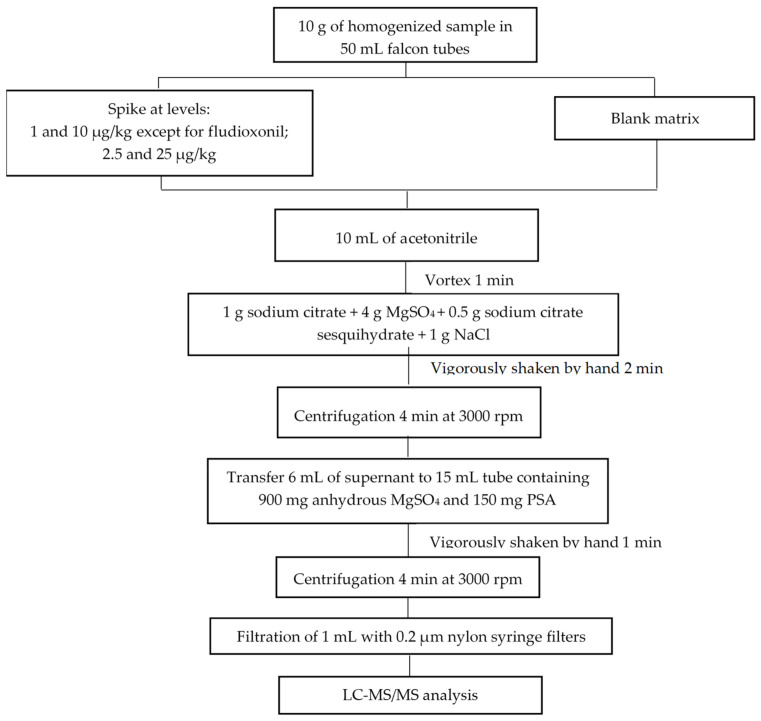
Scheme of the QuEChERS extraction method.

A total of 10 mL acetonitrile was added, and the tube was vigorously shaken for 1 min using a vortex mixer. Afterwards, to induce phase separation and pesticide partitioning, a buffer-salt mixture consisting of 1 g sodium citrate, 4 g MgSO_4_, 0.5 g sodium citrate sesquihydrate and 1 g NaCl was added. The mixture was shaken vigorously by hand for 2 min and centrifuged for 4 min at 3000 rpm. The supernatant phase (6 mL) was subjected to a clean-up dispersive SPE step and pipetted into a 15 mL tube that contained 900 mg anhydrous MgSO_4_ and 150 mg PSA. The tube was vigorously shaken manually for 1 min. Next, centrifugation was carried out for 4 min at 3000 rpm. Finally, 1 mL of the upper acetonitrile layer was filtered through a 0.20 µm nylon filter and transferred to a chromatography vial where 10 μL of the IS solution was added prior to injection into the liquid chromatographic system. In Figure 2, a scheme of the validated extraction method is depicted.

Regarding field samples, the weight of each of the samples received was approximately 3 kg. Each specimen from the sample was chopped into quarters while frozen, and 1 kg was randomly selected and homogenized in the food processor described above. After appropriate homogenization of each sample, two representative subsamples were taken. The samples were analyzed following the previously developed analytical method.

#### 3.3.3. HPLC-MS/MS Analysis

The HPLC-MS/MS system (Agilent Technologies, Santa Clara, CA, USA) consisted of a 1200 Series liquid chromatograph equipped with a triple quadrupole MS/MS (Agilent 6420). Chromatographic separation was achieved with an Atlantis T3 octadecyl silica (C18) column (4.6 × 150 mm, 3 µm) (Waters, Milford, MA, USA). The temperature of the column oven was set at 20 °C during all experiments, and samples were held at a constant temperature of 20 °C by an autosampler thermostat. The mobile phase (A) was a mixture of water with 10 mM ammonium formate and 0.1% formic acid, and the mobile phase (B) was acetonitrile. The gradient elution was programmed as follows: 0–2 min, 80% (B); 2–4 min, 80–90% (B); 4–6.5 min, 90% (B). The flow rate was 0.7 mL/min, and the injection volume was fixed at 10 µL.

The detection was achieved using a triple quadrupole system operating in the multiple-reaction monitoring (DMRM) mode. The positive ionization mode was used for boscalid, fluopyram, pyraclostrobin and tebuconazole, and the negative mode was used for fludioxonil. The ESI source conditions were as follows: gas temperature, 300 °C; drying gas flow rate, 11 L/min; nebulizer pressure, 40 psi; capillary voltage, 4000 V, with N_2_ gas used as the nebulizer gas. Parameters were optimized by injection of a standard fungicide solution of 50 µg/mL of each compound at a flow rate of 0.7 mL/min. Solutions were prepared in 100% acetonitrile. Full–scan spectra were acquired first to optimize the collision-induced dissociation fragmentation applied at the source via the fragmentor voltage to obtain the maximum sensitivity for the precursor ion. Second, MS/MS spectra in the product ion mode of operation were acquired to obtain information on fragment ions. Once the two product ions had been selected for every compound, an MRM (multiple reaction monitoring) experiment was carried out to select the optimum collision energy for each specific transition (Figure 1). Data processing was performed with Agilent Mass Hunter Data Acquisition software (version B.07.00) and processed with Agilent Mass Hunter Quantitative Analysis software (version B.07.00).

#### 3.3.4. Validation Study

The analytical performance was estimated through the determination of selectivity, linearity, limits of detection, limits of quantification, accuracy and precision according to the SANCO guidelines 3029/99 [21], 825/00 [17] and SANTE guidance 11813/2017 [22].

The calibration standards were prepared in matrix-matched solutions in which standards and the internal standard were added to the blank extracts. A total of 7 calibration concentrations ranging between 0.7 and 30 μg/kg were analyzed to establish calibration curves. For comparison purposes, calibration standards in acetonitrile solvent were also prepared. The acceptance criteria were that the correlation coefficient (R^2^) was higher than 0.99, and the linearity residuals were lower than 30%. The accuracy and the precision were obtained by analyzing analytes in blank persimmon samples spiked at two concentration levels, the LOQ and 10× the LOQ, and were evaluated within 1 day in quintuplicate at each concentration level. The acceptance criterion for accuracy was that the recovery fit between 70 and 110%, and for precision, the acceptance criterion was that the relative standard deviation (RSD) was lower than 20%. Selectivity was determined on blank samples of persimmon, which was extracted and analyzed to determine the specificity of the method and to search for interfering peaks under the same conditions, monitoring the selected ion chromatogram characteristic for each analyte. The limit of detection (LOD) was estimated for a signal-to-noise ratio of 3 from the chromatograms at the lowest analyte concentration tested. The limit of quantitation (LOQ) was established as the lowest concentration tested that gave acceptable recoveries and precision.

### 3.4. Residue Extrapolation Proposal

For the residue extrapolation proposal, the first step was to compare the residue level in both crops using the MRL. Secondly, in order to ensure no risk for the consumers, the consumption data of apple and persimmon has been compared.

The data packages of residue levels in persimmons generated in field trials were used for the calculation of MRL and were compared to MRL in apples reported in the reasoned opinions of EFSA [27,28,29,30]. Persimmon and apple MRLs were calculated for each active substance/commodity combination with the internationally adopted OECD MRL Calculator [23]. This statistical tool produces an MRL proposal in the region of the 95th percentile of the underlying residue distribution. The final MRL proposed is the calculated maximum value of three different calculations: (1) the maximum of the highest residue; (2) mean + 4*SD; and (3) 3*mean*CF (the correction factor “CF” is equal to 1 − (⅔ of number censored data (data below LOQ)).

For the intake calculation, the average consumption data from the Pesticide Residue Intake Model EFSA PRIMO rev 3.1 [24] has been considered. In this tool, consumption datasets for the general population and for more vulnerable sub-groups of the population (infants, toddlers, children and vegetarian consumers) are available for several member states of the European Union and other international diets (Table 6). The ratio of the average intake (g/kg body weight/day) of apples/persimmons for each diet was used.

## 4. Conclusions

In this work, results of persimmon residue trials of boscalid, pyraclostrobin, fludioxonil, fluopyram and tebuconazole, as representative pesticides, and existing data from apples as a major crop were used to calculate the MRL ratio and carry out a dietary exposure risk assessment in order to propose a new MRL extrapolation.

In this sense, a simple, reliable and accurate multi-residue method was optimized and validated for the simultaneous determination of pesticides in persimmon fruit based on QuEChERS in combination with HPLC-ESI-MS/MS. The LOQ for all the compounds was 1.0 µg/kg, except for fludioxonil, for which it was 2.5 µg/kg.

Residue levels at harvest of the five representative active substances in four persimmon field trials are consistently below the residue levels determined on apples using identical GAPs in both crops. MRLs were set at 0.5 mg/kg for fludioxonil, 0.6 mg/kg for boscalid, 0.3 mg/kg for tebuconazole, 0.4 mg/kg for fluopyran and 0.3 mg/kg for pyraclostrobin. The MRL ratio (apple/persimmon) was between 2.50 and 1.25 for the pesticides studied. In addition, the European dietary intake contribution of persimmon vs. apple is always well below this, and therefore, consumer safety is guaranteed. Therefore, residue extrapolation from the surrogate major crop, apple, to the minor crop, persimmon, is fully supported and recommended for setting MRLs in persimmon. This will permit the availability of pesticides for this important minor crop in Spain and Mediterranean EU Member States.

## Figures and Tables

**Table 1:** Recovery of selected pesticides from different versions of QuEChERS at a concentration of 0.1 µg/kg in persimmon using LC-MS/MS (n = 3).

Compound	Recovery (%) (RSD %)				
	Extraction	Acetate-Buffered	Acetate-Buffered	Citrate-Buffered	Citrate-Buffered
	Clean-Up	PSA + MgSO_4_	PSA + C18 + MgSO_4_	PSA + MgSO_4_	PSA + C18 + MgSO_4_
Fludioxonil		92.8 (4.1)	102.5 (4.2)	94.6 (4.8)	96.9 (5.7)
Boscalid		102.2 (5.4)	101.6 (4.5)	95.7 (6.2)	95.0 (4.6)
Tebuconazole		92.3 (5.8)	99.4 (7.5)	95.6 (4.6)	97.2 (6.0)
Fluopyram		90.8 (10.2)	101.8 (8.7)	96.4 (5.0)	98.2 (4.9)
Pyraclostrobin		101.2 (8.4)	103.1 (5.1)	98.9(6.1)	94.6 (5.3)

**Table 2:** Molecular weight (M.W.), retention time (R.T.), ionization mode, MRM transitions, and MS operating parameters optimized for analysis of the selected pesticides.

Compound	MW	RT (min)	Ionization Mode	Transition 1 (*m/z*)	DP ^a^ (V)	CE ^b^ (eV)	Transition 2 (*m/z*)	DP ^a^ (V)	CE ^b^ (eV)
Fludioxonil	248.2	4.06	Negative	266 ˃ 229	80	4	266 ˃ 158	80	36
Fluopyram	396.1	4.41	Positive	397 ˃ 173	110	33	397 ˃ 208	110	20
Boscalid	343.2	4.59	Positive	343 ˃ 307	110	17	343 ˃ 271.1	110	32
Tebuconazole	307.8	4.84	Positive	308 ˃ 70	100	21	308 ˃ 124.8	100	41
Pyraclostrobin	387.8	6.45	Positive	388.1 ˃ 194.1	90	8	388.1 ˃ 163	90	25

^a^ Declustering Potential. ^b^ Collision Energy.

**Table 3:** Linear ranges, coefficients of determination (R^2^), detection and quantification limits for the studied compounds and matrix effect.

Compound	Linear Range (µg/kg)	R^2^	LOD ^a^ (µg/kg)	LOQ ^b^ (µg/kg)	Matrix Effect Ss/Sm ^c^
Fludioxonil	1.0–30	0.997	3.8 × 10^−1^	2.5	0.77
Boscalid	0.7–15	0.998	3.1 × 10^−2^	1.0	0.98
Tebuconazole	0.7–15	0.999	4.3 × 10^−2^	1.0	1.01
Fluopyram	0.7–15	0.999	7.0 × 10^−3^	1.0	1.29
Pyraclostrobin	0.7–15	0.999	2.3 × 10^−2^	1.0	1.24

^a^ Limit of detection. ^b^ Limit of quantification. ^c^ Solvent slope/matrix slope.

**Table 4:** Recovery (n = 5), repeatability and intra- and interday precision data for the studied pesticides in fortified persimmon samples.

Compound	Fortification Level (µg/kg)	Mean Recovery (%)	RSD (%)	Intraday RSD ^a^ (%)	Interday RSD ^b^ (%)
Fludioxonil	2.5	93.05	8.19	5.73	4.65
	25.0	99.26	6.89	4.64	2.54
Boscalid	1.0	91.08	6.02	6.19	4.95
	10.0	96.03	9.55	2.41	3.48
Tebuconazole	1.0	102.01	4.88	4.67	6.34
	10.0	94.64	7.31	2.46	4.25
Fluopyram	1.0	93.09	6.13	3.48	3.74
	10.0	87.65	9.57	2.63	2.19
Pyraclostrobin	1.0	89.04	4.70	3.48	6.57
	10.0	91.86	7.91	2.08	4.05

^a^ Intraday relative standard deviation (n = 6). ^b^ Interday relative standard deviation (n = 3).

**Table 5:** Mean concentrations (mg/kg) and relative standard deviation (%) of the target pesticides analyzed in persimmon samples.

Trial ^a^	Plot ^b^	Sample Time ^c^	Pesticide Residue ^d^				
			Boscalid	Pyraclostrobin	Fludioxonil	Fluopyram	Tebuconazole
1	A	0DALA	n.d.	n.d.	n.d.	n.d.	n.d.
		Harvest	n.d.	n.d.	n.d.	n.d.	n.d.
	B	0DALA	0.194 (1.89)	0.095 (0.98)	n.d.	n.d.	n.d.
		Harvest	0.180 (2.18)	0.089 (0.52)	n.d.	n.d.	n.d.
	C	0DALA	n.d.	n.d.	0.171 (0.82)	n.d.	n.d.
		Harvest	n.d.	n.d.	0.099 (1.14)	n.d.	n.d.
	D	0DALA	n.d.	n.d.	n.d.	0.069 (1.47)	0.056 (2.18)
		Harvest	n.d.	n.d.	n.d.	0.052 (2.27)	0.028 (2.81)
2	A	0DALA	n.d.	n.d.	n.d.	n.d.	n.d.
		Harvest	n.d.	n.d.	n.d.	n.d.	n.d.
	B	0DALA	0.239 (1.56)	0.127 (2.21)	n.d.	n.d.	n.d.
		Harvest	0.150 (2.04)	0.066 (0.73)	n.d.	n.d.	n.d.
	C	0DALA	n.d.	n.d.	0.273 (2.60)	n.d.	n.d.
		Harvest	n.d.	n.d.	0.193 (1.72)	n.d.	n.d.
	D	0DALA	n.d.	n.d.	n.d.	0.288 (1.42)	0.273 (1.69)
		Harvest	n.d.	n.d.	n.d.	0.129 (1.18)	0.081 (2.02)
3	A	0DALA	n.d.	n.d.	n.d.	n.d.	n.d.
		Harvest	n.d.	n.d.	n.d.	n.d.	n.d.
	B	0DALA	0.370 (0.82)	0.154 (2.25)	n.d.	n.d.	n.d.
		Harvest	0.173 (1.23)	0.078 (3.04)	n.d.	n.d.	n.d.
	C	0DALA	n.d.	n.d.	0.252 (2.70)	n.d.	n.d.
		Harvest	n.d.	n.d.	0.177 (1.62)	n.d.	n.d.
	D	0DALA	n.d.	n.d.	n.d.	0.187 (0.73)	0.156 (2.90)
		Harvest	n.d.	n.d.	n.d.	0.180 (0.55)	0.119 (1.39)
4	A	0DALA	n.d.	n.d.	n.d.	n.d.	n.d.
		Harvest	n.d.	n.d.	n.d.	n.d.	n.d.
	B	0DALA	0.255 (0.35)	0.097 (1.16)	n.d.	n.d.	n.d.
		Harvest	0.224 (0.60)	0.087 (1.58)	n.d.	n.d.	n.d.
	C	0DALA	n.d.	n.d.	0.223 (2.23)	n.d.	n.d.
		Harvest	n.d.	n.d.	0.096 (1.75)	n.d.	n.d.
	D	0DALA	n.d.	n.d.	n.d.	0.347 (2.54)	0.326 (1.07)
		Harvest	n.d.	n.d.	n.d.	0.170 (1.12)	0.137 (2.81)

^a^ Trials: 1 and 2 orchards in Seville; 3 and 4 orchards in Valencia. ^b^ Pesticides applied in the plots (A: control; B: Bellis (boscalid and pyraclostrobin); C: Geoxe (fludioxonil); D: Luna Experience (fluopyram and tebuconazole)). ^c^ 0DALA: 0 days after last application. ^d^ n.d.: not detected.

**Table 6:** MRL calculations of persimmons and apples and ratio for both crops.

	Persimmon ^a^				Apple ^b^				Apple/Persimmon
Compound	Highest Residue	Mean + 4 SD	CF × 3 Mean	Rounded MRL_OECD_ Calculated ^a^ (mg/kg)	Highest Residue	Mean + 4 SD	CF × 3 Mean	Rounded MRL_OECD_^b^ (mg/kg)	Ratio MRL_OECD_
Fludioxonil	0.19	0.35	0.42	0.50	0.42	0.61	0.78	0.80	1.60
Boscalid	0.22	0.31	0.55	0.60	0.86	1.19	1.27	1.50	2.50
Tebuconazole	0.14	0.28	0.27	0.30	0.21	0.30	0.40	0.40	1.33
Fluopyram	0.18	0.37	0.40	0.40	0.23	034	0.41	0.40	1.00
Pyraclostrobin	0.09	0.12	0.24	0.30	0.29	0.52	0.43	0.60	2.00

^a^ Maximum residue levels calculated with data from Table 4. ^b^ Maximum residue levels from EFSA opinions. SD: standard deviation. CF: correction factor CF= 1 − (⅔ of number censored data (data below LOQ)).

**Table 7:** Comparison of the average consumption data-food intake (g/kg body weight/day) apple-persimmon for the EU Member States.

Average Consumption Data-Food Intake (g/kg Body Weight/day)				
Diet for Chronic Exposure	Subgroup of Population/Age Group	Persimmon	Apple	Ratio Apple/Persimmon
DE child	Children between 2–5 years	0.0100	12.48	1248.0
DE general	General population	0.0088	2.43	275.3
DE women	Women of child-bearing age	0.0139	2.58	185.6
DK adult	15–74 years	0.0027	0.96	350.1
DK child	4–6 years	0.0000	2.32	-
ES adult	Adults ≥ 17 years	0.0100	0.77	76.9
ES child	7–12 years	0.0041	1.14	278.6
FI adult	Adults	0.0000	0.94	-
FI child	Children up to 6 years	0.0000	0.58	-
FR infant	7–18 months	0.0000	1.68	-
FR toddler	25–36 months	0.0000	3.17	-
FR child	Children from 3 to <15 years	0.0000	1.68	-
FR adult	Adults ≥ 15 years	0.0000	0.77	-
IE adult	Adults 18–64 years	0.0000	0.71	-
IE child	5–12 years	0.0000	0.33	-
IT adult	18–64 years	0.0000	0.79	-
IT toddler	1–17 years	0.0000	0.89	-
LT adult	19–64 years	0.0000	1.87	-
NL child	2–6 years	0.0000	5.78	-
NL general	General population, 1–97 years	0.0000	1.46	-
NL toddler	8 to 20 months	0.0000	10.78	-
PL general	General population, 1–96 years	0.0000	2.04	-
PT general	General population	0.0000	1.05	-
RO general	General population	0.0000	1.42	-
SE general	General population, 1–74 years	0.0000	1.05	-
UK infant	6 months–1 year	0.0000	1.56	-
UK toddler	18 months–4 years	0.0000	1.71	-
UK adult	19–64 years	0.0000	0.41	-
UK vegetarian	No information	0.0000	0.59	-
* GEMS/Food G06	General population	0.0042	0.92	221.9
GEMS/Food G07	General population	0.0017	1.02	614.4
GEMS/Food G08	General population	0.0050	1.21	242.7
GEMS/Food G10	General population	0.0025	0.75	301.2
GEMS/Food G11	General population	0.0017	1.55	932.8
GEMS/Food G15	General population	0.0017	1.09	656.3

Source: EFSA PRIMO rev 3.1. * GEMS: Global Environment Monitoring System. Food Cluster diets relevant for the EU Member States (i.e., Cluster diet G06, G07, G08, G10, G11 and G15).

## Data Availability

Not applicable.

## Sample Availability

Samples of the compounds are available from the authors.
